# Facilitated DNA damage repair as an emerging therapeutic strategy for inflammatory and fibrotic diseases

**DOI:** 10.1039/d5cb00327j

**Published:** 2026-03-23

**Authors:** Maurice Michel, Nayere Taebnia, Volker M. Lauschke

**Affiliations:** a Center for Molecular Medicine, Karolinska Institutet and University Hospital SE-171 77 Stockholm Sweden maurice.michel@ki.se; b Science for Life Laboratory, Department of Oncology-Pathology, Karolinska Institutet Stockholm Sweden; c Department of Physiology and Pharmacology, Karolinska Institutet SE-171 77 Stockholm Sweden volker.lauschke@ki.se +46 852487711; d Dr Margarete Fischer-Bosch Institute of Clinical Pharmacology Stuttgart Germany; e University of Tübingen Tübingen Germany; f Department of Pharmacy, the Second Xiangya Hospital, Central South University Changsha China

## Abstract

DNA damage arising from metabolic stress, oxidative injury, and impaired genome maintenance emerges as a common driver for chronic inflammatory and fibrotic diseases across multiple organs. While rapid and effective DNA damage repair is essential for the response to acute injury, sustained activation of these pathways promotes cellular senescence, sterile inflammation and fibroblast activation, ultimately driving fibrogenesis and pathological tissue remodelling. In recent years, DNA repair processes, particularly base excision repair in both the nucleus and mitochondria, receive increasing attention as modulators of inflammatory and fibrotic outcomes. Here, we review the molecular mechanisms by which unresolved nuclear and mitochondrial DNA lesions translate into chronic inflammation and fibrosis across skin, liver, lung and cardiovascular tissues. We discuss the roles of chromatin context, NAD^+^ availability, repair intermediates and mitochondrial genome instability in shaping DNA damage responses and highlight emerging chemical biology strategies to facilitate DNA repair, including organocatalytic switches of DNA glycosylases, DNA polymerase γ (POLG) activators or small molecules targeting the inflammasome or cGAS–STING pathway. Based on the available evidence from animal models and organotypic human *in vitro* cultures, we propose that facilitated DNA repair may represent a promising therapeutic strategy for chronic inflammatory and fibrotic diseases. This perspective positions genome maintenance pathways as upstream intervention points for chronic inflammatory and fibrotic diseases.

## Abstract 2

Persistent nuclear and mitochondrial DNA damage links metabolic stress to inflammation and fibrosis. Facilitating DNA repair pathways may restore genome stability and limit fibrotic remodeling across tissues.

## Graphical abstract

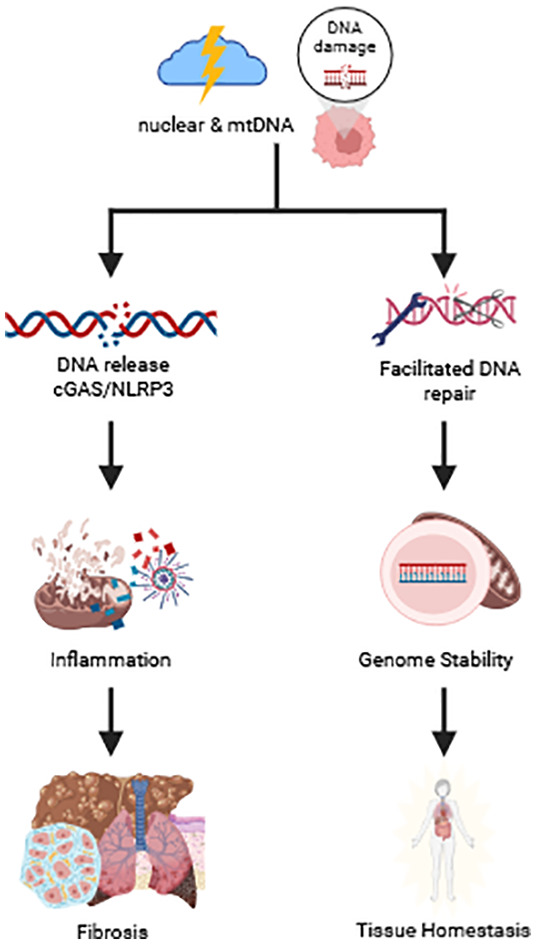

## 1. Introduction

Fibrosis is a common feature of many chronic inflammatory diseases across organs, including liver, lung, skin and heart and remains a major cause of morbidity and mortality worldwide. Despite diverse etiologies, fibrotic disorders share various hallmarks, including persistent inflammation, cellular senescence, fibroblast activation and an excessive buildup of extracellular matrix (ECM) that replaces the functional parenchyma. Current anti-fibrotic therapies primarily target downstream inflammatory or pro-fibrotic signalling pathways with limited efficacy regarding the reversal of the disease.^1^

An emerging body of evidence indicates that persistent DNA damage drives chronic inflammation and fibrosis.^2,3^ Metabolic stress and mitochondrial dysfunction result in the formation of elevated levels of reactive oxygen species (ROS), which generates oxidative DNA damage lesions, particularly 8-oxo-7,8-dihydroguanine (8-oxoG). When the load of DNA lesions exceeds the capacity of repair pathways, DNA damage response (DDR) signalling is activated, which promotes cellular senescence, stimulates pro-inflammatory signaling and activates fibroblasts, which jointly shape a pro-fibrotic microenvironment.^4,5^ Importantly, these lesions are not limited to oxidized bases but also include bulky adducts, replication-associated lesions, DNA double-strand breaks and telomere-associated damage, which can similarly drive persistent DDR signaling and senescence.^6^

In this review, we summarize the role of DNA damage repair across organ systems, including the skin, liver, lung and cardiovascular system, with a focus on metabolic diseases and aging. We present nuclear and mitochondrial repair pathways and their integration with innate immune sensing and highlight emerging pharmacological approaches to provide repair directionality. Lastly, we discuss how facilitated DNA damage repair can emerge as an actionable strategy to counteract chronic inflammation and fibrosis.

## 2. Molecular mechanisms of oxidative DNA damage facilitators

### 2.1 Genomic DNA damage repair

Oxidative stress constitutes a ubiquitous source of DNA damage and among oxidative lesions, 8-oxo-7,8-dihydroguanine (8-oxoG) is the most common.^6^ In recent years research has established that 8-oxoG removal and coordination of downstream repair determine whether cells resolve damage, activate inflammatory signalling, undergo senescence or enter fibrotic remodelling.^7–9^ While the focus has historically been on 8-oxoGuanine DNA glycosylase 1 (OGG1) as the primary enzyme responsible for 8-oxoG removal, a broader set of DNA glycosylases and nuclear regulators define the DNA repair outcome,^10^ influencing inflammation and fibrosis risk.

One out of eleven human DNA glycosylases initiate base excision repair (BER), the DNA repair pathway that predominantly removes nucleobases damaged by oxidation. While evolution does not generally favour redundancy in core repair reactions, a number of DNA glycosylase exhibit overlapping substrate specificity.^11^ This supports a model in which the individual enzymes contribute to roles in different cellular compartments and during distinct cell and even life cycle. In addition, some of the enzymes support each other's function through establishing a second line of repair, especially when the removal of lesions is challenged or delayed.

For example, mutY DNA glycosylase (MUTYH) removes adenine that mis-paired with 8-oxoG during replication, thereby controlling the mutagenic G → T transversions.^12^ Under conditions where OGG1 fails or is inhibited, MUTYH activity may instead generate abasic (AP) sites on the complementary strand, potentially leading to strand breaks, replication stress and persistent DNA damage signalling. This mechanism contributes to senescence or cell death rather than clean repair. Indeed, in specific contexts such as neurons, MUTYH-driven repair plays a functional role in neurodegeneration under oxidative load.^13^

Endonuclease VIII-like proteins 1, 2 and 3 (NEIL1-3), as well as endonuclease III-like protein (NTHL1) recognize a broader spectrum of oxidized bases, including oxidized pyrimidines, ring-opened purines, hydantoins, and other variants often generated by further oxidation of 8-oxoG or clustered damage.^14,15^ NEIL1 and NEIL2 have been shown in cell-based studies to accumulate at DNA damage sites especially when OGG1 is absent or inhibited, suggesting compensatory backup within BER.^15^ However, when the load of DNA lesions is high, the downstream BER cascade can become overwhelmed. This can result in competition between glycosylases, leading to unscheduled AP site formation, inefficient hand-off, and persistent repair intermediates. This, in turn, may trigger inflammatory signalling, replication stress and senescence.^16^

Other monofunctional glycosylases, *e.g.* the single-strand selective monofunctional uracil DNA glycosylase (SMUG1) and uracil DNA glycosylase (UNG) as well as alkyl adenine DNA glycosylase (AAG), contribute further to nucleobase repair. However, under high endogenous or exogenous stress, their combined activity can overload BER capacity with AP sites, risking accumulation of unrepaired lesions or more toxic repair intermediates further downstream.^17,18^

Importantly, the repair outcome is not determined solely by the presence, abundance and stoichiometry of DNA glycosylases. In addition, nuclear context, chromatin state, scaffold recruitment and signalling determine repair efficiency, inflammatory output, and risk of fibrogenesis.^19^ Here, chromatin modifiers, such as histone deacetylase 1 and 3 (HDAC1 and HDAC3), modulate chromatin accessibility. Evidence indicates that compact, hypoacetylated chromatin reduces access of DNA glycosylases and downstream BER enzymes, slowing repair and increasing lesion persistence.^20^ In contrast, open chromatin facilitates efficient repair. Under chronic oxidative load, increased HDAC activity or insufficient histone acetylation may reduce BER throughput, increasing inflammation and fibrosis risk.^21–23^

Beyond chromatin accessibility itself, deacetylases, among them the sirtuins SIRT1 and SIRT6 as well as HDAC1, regulate multiple layers in DNA repair. Deacetylation of repair enzymes like glycosylases and apurinic/apyrimidinic endonuclease 1 (APE1) up- and downregulate enzymatic function.^24,25^ When targeted by deacetylases, histones loosen chromatin, and transcription factors, such as NF-κB, modulate inflammatory output. Reduced sirtuin activity, which constitutes a common hallmark of aging and metabolic stress, may slow repair, sustain inflammatory signalling, and promote senescence-fibrosis transition. Indeed, pharmacological modulation of sirtuins alters repair efficiency and inflammatory gene expression in oxidatively stressed cells.^26^

Another complex important for facilitated oxidative DNA damage repair consists of X-ray repair cross-complementing protein 1 (XRCC1) as a repair scaffold and poly(ADP-ribose)-polymerase 1 (PARP1) as a sensor of DNA strand breaks. Efficient PARP1–XRCC1 recruitment is essential for completing BER and restoring integrity. However, excessive PARP1 activation due to high lesion load or inefficient repair can cause nicotinamide adenine dinucleotide (NAD^+^) depletion, energetic failure, cell death or chronic inflammation. These processes contribute to fibrotic remodelling. Studies have demonstrated that NEIL1 and OGG1 interact with PARP1, and that defects in the proteins impair PARP1-mediated poly(ADP-ribosyl)ation, reducing repair capacity in aged cells.^27,28^

In addition, dual-function kinases, such as ataxia-telangiectasia mutated (ATM), ataxia telangiectasia and Rad3-related protein (ATR) but also DNA-dependent protein kinase (DNA-PK) play key roles in cell cycle checkpoints and non-homologous end joining, and translate unresolved DNA lesions or repair intermediates into cell-cycle arrest, senescence, or pro-fibrotic gene programs.^29^ Chronic low-level activation of these pathways contributes to stable senescence, senescence-associated secretory phenotype (SASP), myofibroblast activation and ECM deposition converting damage burden into a fibrotic phenotype.^30,31^ It is therefore important to interpret facilitated nuclear DNA repair as a networked system, consisting of lesion sensors like DNA glycosylases, chromatin state regulators, repair scaffolds and checkpoint transducers that only collectively determine the outcome.

Besides oxidative base lesions, additional forms of DNA damage are also relevant to chronic inflammation and fibrosis.^4^ Bulky DNA adducts and UV photoproducts require nucleotide excision repair (NER), and persistence of such lesions can sustain ATR-dependent checkpoint signaling and inflammatory gene expression.^32^ Likewise, replication stress and transcription–replication conflicts can lead to stalled forks, accumulation of single stranded DNA and collapse into DNA double-strand breaks, thereby engaging ATM, ATR and DNA-PK signaling.^29,33,34^ Telomere-associated damage is particularly important in chronic degenerative disease, as telomeric lesions are repaired inefficiently and can trigger persistent DDR signaling, cellular senescence and fibrotic remodeling even in the absence of widespread genomic instability.^35,36^ Across these lesion classes, a common consequence is prolonged checkpoint activation, chromatin remodeling, SASP and innate immune activation, all of which converge at the level of fibroblast activation and ECM remodeling.

More globally, recent work identified the DREAM complex as a conserved transcriptional repressor of somatic DNA repair capacity across multiple repair pathways, supporting the concept that fibrotic phenotypes may emerge not only from individual repair defects but also from system-level restriction of genome maintenance programs.^37^ Thus, while BER represents the most developed therapeutic entry point at present, fibrosis should be viewed more broadly as a consequence of unresolved genome instability across multiple DNA damage response pathways.

### 2.2 Mitochondrial DNA damage repair

**Fig. 1:**
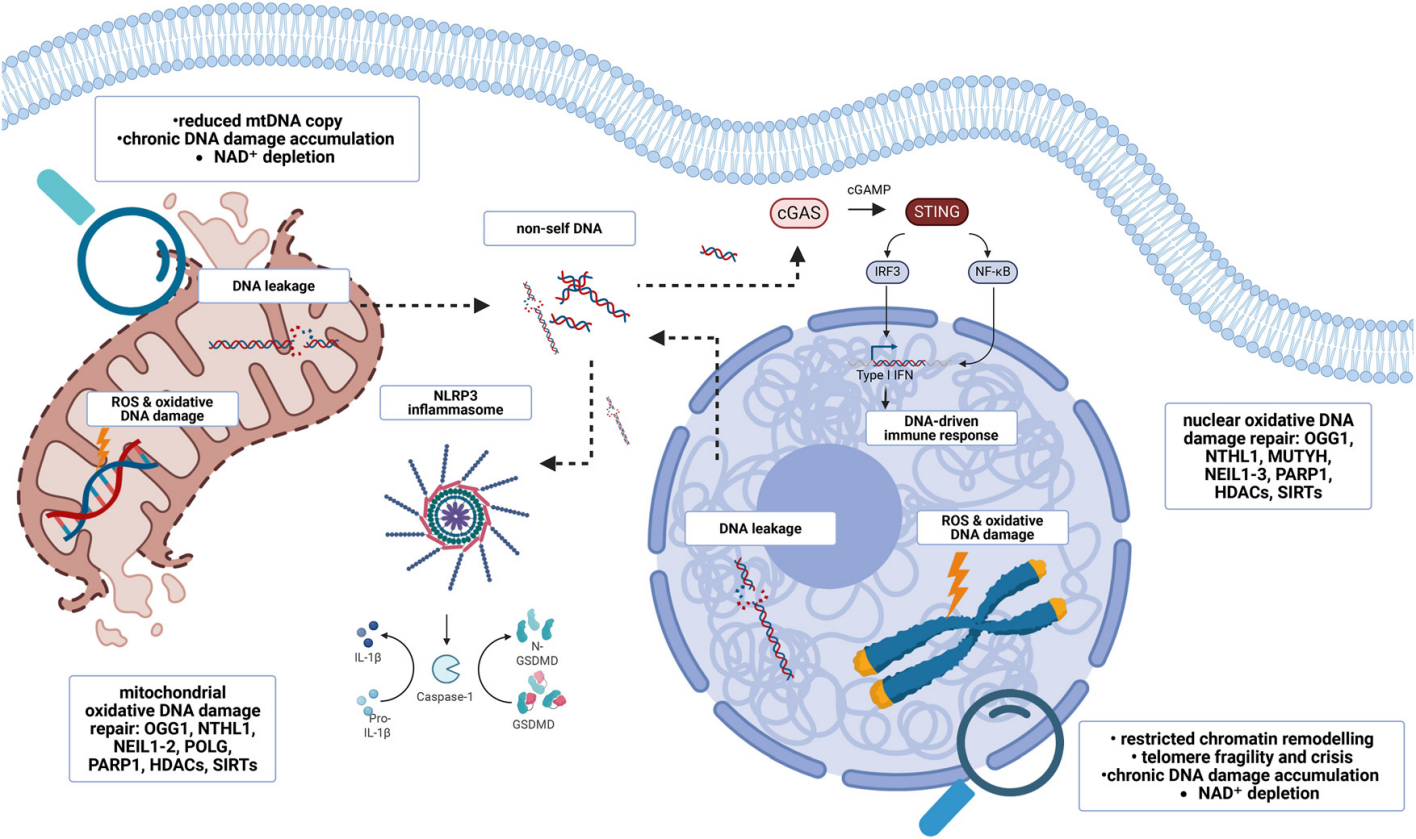
DNA damage-driven inflammatory signaling links nuclear and mitochondrial genome instability to fibrosis. Oxidative stress induces DNA damage in both the nuclear and mitochondrial compartments, generating lesions such as 8-oxoG that are processed by base excision repair (BER) pathways. In the nucleus, inefficient or mis-coordinated repair involving OGG1, NTHL1, MUTYH, NEIL1-3, PARP1, histone deacetylases (HDACs) and sirtuins can lead to restricted chromatin remodeling, telomere fragility, NAD^+^ depletion and chronic DNA damage accumulation, promoting senescence and DNA leakage. In mitochondria, limited redundancy of the mtDNA repair machinery (OGG1, NTHL1, NEIL1–2, POLG, PARP1, HDACs and sirtuins) results in reduced mtDNA copy number, persistent damage and release of oxidized mtDNA into the cytosol. Cytosolic DNA activates innate immune sensors, including cGAS–STING, triggering type I interferon and NF-κB-dependent inflammatory gene expression, as well as the NLRP3 inflammasome, leading to caspase-1 activation, gasdermin D cleavage and IL-1β release. Together, unresolved nuclear and mitochondrial DNA damage converges on chronic inflammation, fibroblast activation and fibrotic tissue remodeling.

**Fig. 2:**
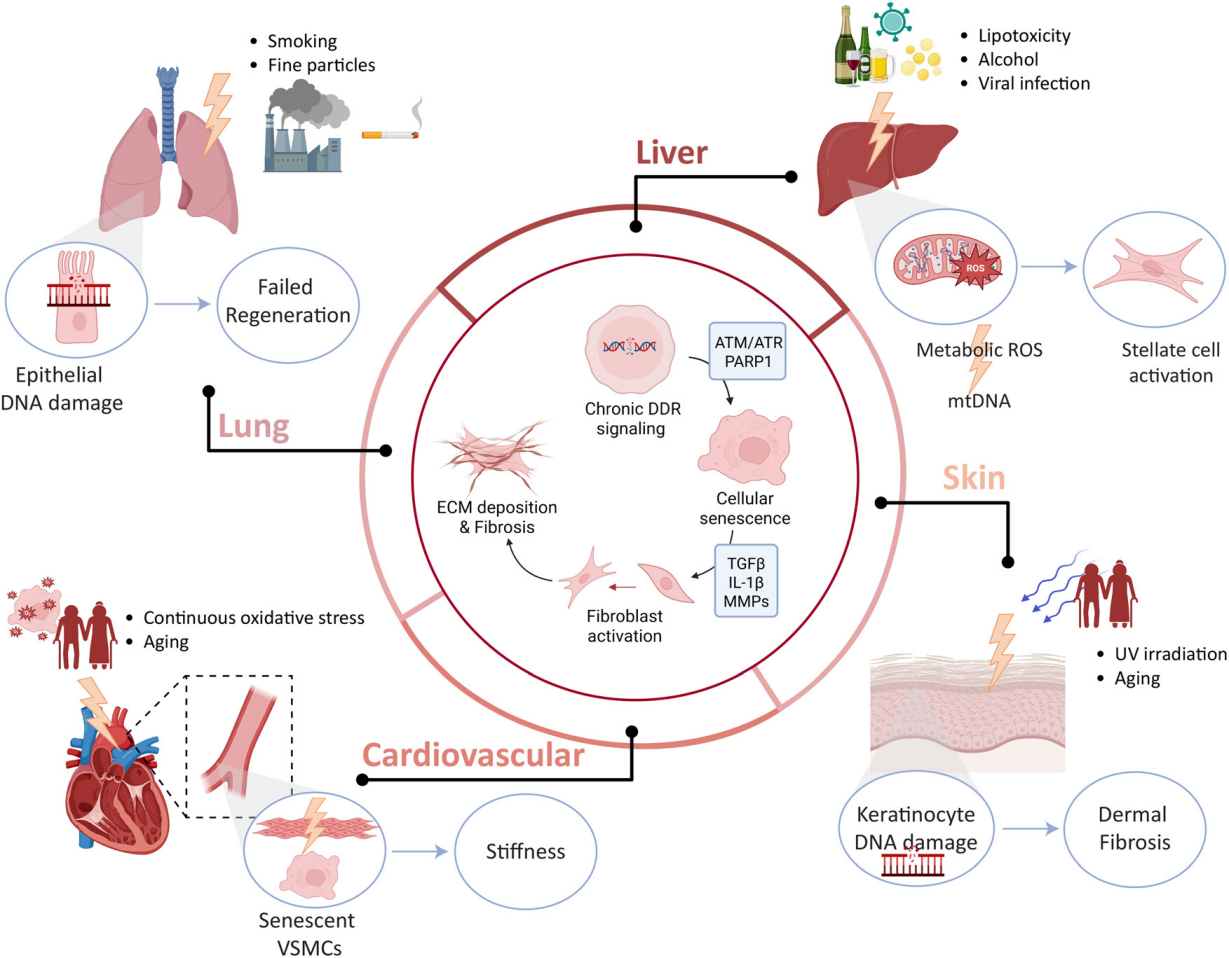
Persistent DNA damage as a unifying theme that links organ-specific stressors to fibrosis across organs. Organ-specific environmental, metabolic, and mechanical stressors induce nuclear and mitochondrial DNA damage in skin, liver, lung, and cardiovascular tissues. In the lung, inhaled toxins, smoking, and fine particulate matter promote epithelial DNA damage, impaired regeneration, and fibroblast activation. In the liver, lipotoxicity, alcohol exposure, and viral infection drive the formation of reactive oxygen species (ROS), mitochondrial DNA (mtDNA) damage, and activation of hepatic stellate cells. In the skin, ultraviolet irradiation and aging induce keratinocyte DNA damage, triggering dermal fibroblast activation and cutaneous fibrosis. In the cardiovascular system, sustained oxidative stress and aging promote senescence of vascular smooth muscle cells (VSMCs), contributing to vessel stiffening. Despite distinct initiating insults, chronic DNA damage, defective repair, cellular senescence and sustained DDR signaling converge on shared downstream outcomes, including inflammatory cytokine production, extracellular matrix deposition and progressive fibrosis across tissues.

Mitochondria harbor their own genome in the form of mitochondrial DNA (mtDNA), which, compared to nuclear DNA, is more vulnerable, especially to oxidative damage. The combination of high reactive oxygen species (ROS) exposure from the electron transport chain, proximity of mtDNA to ROS sources, open chromatin configuration and continuous replication renders mtDNA especially susceptible to oxidative lesions such as 8-oxoG.^38^ This structural vulnerability places mitochondrial genome maintenance at a critical junction where efficient repair mechanisms are required to preserve cellular homeostasis. When repair capacity is compromised, mitochondrial DNA damage can accumulate and activate inflammatory pathways that contribute to fibrotic tissue remodeling (Fig. 1 and 2).^39,40^

Mitochondrial base excision repair (mtBER) represents the principal pathway responsible for the removal of oxidized lesions in mtDNA. Experimental studies using biochemical assays and genetic models have demonstrated that several nuclear DNA glycosylases are also targeted to mitochondria, where they initiate lesion recognition and excision.^41,42^ In particular, the mitochondrial isoform of OGG1, as well as glycosylases such as NEIL1 and NEIL2, have been shown to recognize and remove oxidized bases such as 8-oxoG from mtDNA.^43–45^ Subsequent processing of AP sites is primarily mediated by APE1, which cleaves the DNA backbone to generate single-strand breaks that serve as substrates for repair synthesis.^46^ DNA synthesis during mtBER is carried out by the mitochondrial DNA polymerase POLG, the sole polymerase responsible for mtDNA replication and repair, while ligation of the repaired strand is typically mediated by DNA ligase III (LIG3).^47,48^ Because mitochondrial DNA maintenance relies largely on a single polymerase and ligase, mtBER exhibits considerably less redundancy than nuclear DNA repair pathways (Table 1).

**Table 1:** Emerging therapeutic targets linking DNA damage repair to inflammation and fibrosis. BER = base excision repair; DDR = DNA damage repair; SASP = senescence-associated secretory phenotype

Target/pathway	Cellular compartment	Therapeutic rationale	Intervention type	Development status
OGG1	Nuclear DNA	Efficient removal of 8-oxoG prevents persistent DDR signaling, senescence and SASP; context-dependent modulation alters inflammatory output	Enzyme delivery, small-molecule activators, ORCAs; transient inhibitors (acute settings)	Preclinical
OGG1 (mitochondrial)	mtDNA	Removal of oxidized mtDNA limits mtDNA fragmentation, leakage and innate immune activation	Mitochondrial targeting of OGG1; metabolic support (SIRT3/NAD^+^)	Preclinical
POLG	mtDNA	Boosting replication and repair stabilizes mtDNA content and mitochondrial function	Small-molecule activators	Phase1/phase 2
BER scaffolds (XRCC1/PARP1)	Nuclear DNA	Efficient BER completion prevents NAD^+^ depletion, energetic stress and chronic inflammation	Indirect modulation *via* repair facilitation and NAD^+^ preservation	Multiple PARP1 inhibitors on market
Sirtuins (SIRT1, SIRT3, SIRT6)	Nuclear & mitochondrial	Deacetylation of repair enzymes and chromatin supports repair flux, suppresses inflammation and senescence	Small-molecule activators; NAD^+^ restoration	Multiple preclinical an early clinical programs
NAD^+^ metabolism	Nuclear & mitochondrial	Sustains sirtuin activity, PARP balance and repair enzyme function under oxidative stress	NAD^+^ precursors, metabolic modulation	Clinical (adjacent indications)
cGAS–STING	Cytosolic DNA sensing	mtDNA leakage activates innate immunity and chronic inflammation	Small-molecule inhibitors	Clinical trials ongoing
NLRP3 inflammasome	Cytosolic innate immune signaling	Oxidized mtDNA amplifies inflammation and fibrosis *via* IL-1β/IL-18	Small-molecule inhibitors (*e.g.* MCC950-class, dapansutrile)	Clinical trials ongoing
TFAM/mitochondrial nucleoids	mtDNA packaging	Stabilization of mtDNA prevents fragmentation and cytosolic escape	Genetic and pharmacological stabilization	Preclinical
Telomere maintenance (TERT, telomere BER)	Nuclear telomeres	Telomeric damage drives senescence and fibrosis; repair restores regenerative capacity	Transient telomerase activation; telomere repair support	Preclinical

Thus, any bottleneck, such as insufficient glycosylase or APE1 activity, inhibition of POLG, compromised ligase or defective mtDNA packaging can rapidly lead to accumulation of mtDNA lesions, fragmentation or loss of copies. Experimental evidence supports that this fragility can have direct consequences for mitochondrial function and cellular homeostasis. In animal models, mitochondrial delivery of human OGG1 reduces oxidative mtDNA damage, normalizes mtDNA content and mitigates metabolic derangements, *e.g.* reduced insulin resistance under high-fat diet conditions.^49^ Mitochondrial overexpression of OGG1 protects against a high-fat diet, supports oxidative phosphorylation and leads to reduced levels of neuroinflammation during aging.^50–52^ In addition, we recently reported on a small molecule strategy to boost mitochondrial OGG1 function, supporting organelle maintenance and morphology.^53^ Further, activation of POLG supports the synthesis of mtDNA, enhances biogenesis of the oxidative phosphorylation machinery and cellular respiration,^54^ reinforcing that stimulation of BER and mitochondrial genome maintenance may play a role in mitochondrial health.

An additional layer of regulation is post-translational control of repair enzymes. For example, the mitochondrial isoform of OGG1 is deacetylated and stabilized by the mitochondrial deacetylase SIRT3.^55^ In cell-based studies, SIRT3-mediated deacetylation of OGG1 prevented its degradation under oxidative stress and maintained its incision activity, thereby protecting mtDNA and preventing apoptotic cell death. The enzymatic activity of SIRT3 depends on NAD^+^ availability, linking cellular and metabolic redox status to repair capacity.^56^ In conditions of metabolic stress, NAD^+^ depletion or SIRT3 downregulation may impair mtBER, reduce lesion clearance, and increase risk of mtDNA release plus inflammatory signalling.^55^ Consistent with this, aging, driven in part by mitochondrial dysfunction, NAD^+^ decline and impaired mitophagy, is increasingly recognized to involve mtDNA-driven inflammation and organ fibrosis.^57^

Thus, mitochondrial DNA repair efficiency is determined not only by repair enzymes but also by mitochondrial quality control, metabolic state and post-translational modifications, which are all modifiable factors with therapeutic relevance. Therefore, in tissues under chronic oxidative or metabolic stress, such as in the liver in metabolic dysfunction-associated steatohepatitis (MASH), the heart under ischemia, a fibrotic lung or an aging vasculature, the capacity and fidelity of mtDNA repair become critical determinants of long-term cell viability and organ remodelling.

### 2.3 MtDNA release and inflammasome activation

When mitochondrial repair fails, mtDNA fragments or whole mitochondrial genomes can be released into the cytosol. Multiple pathways mediate such release, including mitochondrial permeability transition pore (mPTP) opening, voltage-dependent anion channels (VDAC) in the outer mitochondrial membrane, mitochondrial fission/fusion imbalance and impaired mitophagy, and converge at the level of mitochondrial breakdown and leakage of mtDNA into the cytoplasm.^58^ Once released, mtDNA is recognized as a damage-associated molecular pattern (DAMP) and triggers downstream signalling cascades.

#### 2.3.1 cGAS–STING activation upon DNA binding

Cyclic GMP-AMP synthase (cGAS) is a cytosolic double-stranded DNA sensor that normally remains inactive in healthy cells. Oxidized mtDNA fragments, particularly those containing intact double-stranded regions, act as high-affinity cGAS ligands. Upon binding, cGAS produces the second messenger cyclic GMP-AMP (cGAMP), which activates the adaptor protein STING in the endoplasmic reticulum.^58^ Downstream, TBK1 and IRF3 drive type I interferon production, while NF-κB triggers transcription of inflammatory genes, chemokines and tissue remodelling programs. In effect, mtDNA leakage transforms a metabolic defect into a sterile inflammatory response that can persist even after the initial damage resolves.

cGAS–STING activation has now been implicated in fibrosis across different organs.^59^ For instance, mitochondrial dysfunction in macrophages can lead to the release of mtDNA into the cytosol, where it activates cGAS and drives sterile liver injury and inflammation.^58,60^ In ischemia-reperfusion models, leaked mtDNA accumulates in the cytosol and activates cGAS–STING, contributing to neuronal cell death and chronic damage signalling.^61^ Similar patterns have been reported in fibrotic lung and vascular aging,^62,63^ suggesting that cGAS is a general amplifier of mitochondrial injury into systemic inflammation. Importantly, cGAS activation can also occur downstream of nuclear DNA damage. Recent studies demonstrate that unresolved genomic lesions can lead to chromosome missegregation and micronuclei formation, exposing nuclear DNA to cytosolic sensors and thereby activating the cGAS–STING pathway and inflammatory signaling.^64–66^

#### 2.3.2 NLRP3 recognition of oxidized mtDNA

It is established that oxidized mtDNA binds and activates NLR family pyrin domain containing 3 (NLRP3) directly, possibly through an 8-oxoG-binding scaffold.^67^ Activation triggers apoptosis-associated speck-like protein containing a CARD (ASC) recruitment, caspase-1 cleavage, and release of IL-1β and IL-18, cytokines that are strongly associated with fibroblast activation, myofibroblast conversion, ECM accumulation and collagen deposition.^68^ In tissue injury models, inhibiting NLRP3 reduces fibrosis even without altering upstream damage, indicating that NLRP3 acts as a fibrosis signal amplifier downstream of mtDNA leakage.^69^ Thus, mitochondrial genome instability follows a predictable sequence consisting of mtDNA damage, mtDNA leakage, cGAS activation, STING signalling or activation of NLRP3 inflammasome and fibro-inflammatory remodelling. It has become evident in the literature, that even mild mitochondrial dysfunction can produce chronic disease if mtDNA leakage continues.

## 3. Facilitated DNA damage repair for dermatitis and cutaneous fibrosis

The skin is continuously exposed to genotoxic stress, particularly ultraviolet (UV) radiation, environmental pollutants, and oxidative stress. As a result, epidermal and dermal cells accumulate a spectrum of DNA lesions, including cyclobutane pyrimidine dimers (CPDs), and oxidative base damage such as 8-oxoG. Upon efficient repair, skin injury remains transient. However, a dysfunctional response may result in the accumulation of persistent DNA damage and drive chronic inflammation, fibroblast activation, dermal thickening and ultimately cutaneous fibrosis.

In sustained DNA damage signalling, both inflammatory dermatitis and fibrotic skin disorders share a common upstream feature. UV-induced lesions activate ATR/ATM signalling, PARP1,^70^ and p53, leading to keratinocyte cell-cycle arrest, apoptosis or senescence.^71^ Senescent keratinocytes and fibroblasts secrete cytokines, growth factors and matrix-modifying enzymes that remodel the dermal ECM. Over time, this SASP promotes fibroblast activation and collagen deposition, coupling epidermal DNA damage to dermal fibrosis.^72^ Oxidative DNA damage further amplifies these processes. 8-OxoG accumulation triggers BER, with OGG1-dependent repair intermediates contributing to the expression of pro-inflammatory genes when repair is incomplete. Importantly, the skin experiences repeated low-dose injury, making it particularly sensitive to cumulative repair inefficiency rather than acute failures.

The skin provides the clearest and most mature evidence that facilitating DNA repair is therapeutically beneficial. Topical delivery of DNA repair enzymes has been developed to accelerate lesion removal following UV exposure.^73^ Liposome-encapsulated photolyase directly reverses CPDs, reduces UV-induced DNA damage, suppresses inflammatory responses and slows features of photoaging in both preclinical and clinical studies.^74,75^ Similar effects have been reported for topical formulations containing T4 endonuclease V, which initiates repair of CPDs *via* the nucleotide excision repair pathway.^76^ These interventions demonstrate that acceleration of lesion clearance attenuates downstream inflammation and tissue remodelling, even after successful DNA damage repair.

Beyond UV photolesions, oxidative DNA damage is abundant in chronically inflamed or aged skin. OGG1, NEIL1/2 and associated BER enzymes are expressed in keratinocytes and fibroblasts and their activity declines with age and chronic inflammation.^8,77^ Reduced BER capacity correlates with increased oxidative damage burden, prolonged DNA damage response (DDR) signalling, and enhanced fibroblast activation. Further, impaired BER leads to persistent strand-break intermediates, PARP1 hyperactivation, NAD^+^ depletion and cellular dysfunction, processes strongly linked to fibrotic remodelling.^78,79^

Telomeres are particularly vulnerable to oxidative stress and telomeric DNA damage repair is less efficient than in other parts of the chromosome. Accumulation of telomere-associated lesions drives replicative senescence in keratinocytes and fibroblasts, contributing to epidermal thinning, impaired regeneration, and dermal fibrosis.^80^ Importantly, strategies that stabilize telomeres or enhance repair responses at telomeres mitigate skin aging and fibrotic responses. Recent studies employing TERT mRNA delivery or transient telomerase activation in the context of radiation-induced skin injury demonstrated improved tissue repair and reduced fibrotic remodelling.^81^

The dominant injury type in cutaneous fibrosis is chronic and cumulative due to environmental exposure, rather than being due to acute bursts of metabolic stress. This renders the skin a model for enhanced DNA repair, mainly due to limited proliferation compared to other organs. As such, in tissues subjected to repetitive low levels of genotoxic stress, facilitated DNA repair acts through reducing inflammation, minimizing senescence and limiting fibrotic remodelling. This stands in sharp contrast with acute injury mechanisms, where transient suppression of repair-linked inflammatory signalling may be beneficial.

## 4. Facilitated DNA damage repair for hepatitis and hepatic fibrosis

The liver is continuously exposed to genotoxic stress arising from endogenous metabolic activity and exogenous insults. Common stressors include lipotoxicity in MASH, alcohol metabolism, various xenobiotics and hepatotropic viruses that all result in elevated formation of ROS.^82,83^ As in other organs, persistent oxidative stress results in an increased load of 8-oxoG and other DNA damage lesions in the liver.^84,85^ This results in cell-cycle arrest, apoptosis, or senescence, depending on injury severity and repair capacity. Senescent hepatocytes secrete pro-inflammatory cytokines and chemokines. This SASP amplifies immune cell recruitment and directly activates hepatic stellate cells (HSCs), thereby linking metabolic hepatocyte dysfunction to inflammation, stellate cell transdifferentiation and fibrotic ECM remodeling.

Senescence in the liver is also linked to the formation of polyploid hepatocytes and oxidative stress directly favors polyplodization in metabolic liver disease.^86^ Polyploid hepatocytes are less proliferative and thus might confer protection against malignant transformation due to extensive DNA damage.^87^ Polyploidization can thereby buffer against gene-inactivating mutations when DNA damage repair is overwhelmed.^88^ Thus, polyploidization can maintain hepatocyte function while removing the damaged hepatocytes from the proliferative pool.

DNA glycosylases are important modulators of hepatic metabolism and the response to injury. Mice deficient in Neil1, Smug1 or Ogg1 exhibit hyperglycemia, hyperinsulinemia, impaired hepatic mitochondrial function and hepatic steatosis.^89–91^ These results indicate that DNA glycosylases play important roles in hepatic homeostasis. These results are corroborated by previous experimental data showing that small molecule modulators of OGG1 that act as organocatalytic switches (ORCAs) to accelerate the repair of oxidative lesions could reduce fibrosis in *ex vivo* 3D liver cultures derived from MASH patients.^92^ In contrast to DNA glycosylases, PARP appears to be pro-inflammatory and pro-fibrotic.^93–95^ Interestingly, these findings seem to contrast new results that demonstrate that increasing the hepatic NAD^+^ pool by inhibiting aminocarboxymuconate semialdehyde decarboxylase (ACMSD), a key factor in controlling NAD^+^ biosynthesis, ameliorates MASH and fibrosis phenotypes in various mouse models and human organoids.^96^ Further research will be required to consolidate these different observations.

Mitochondrial dysfunction constitutes a central feature of chronic liver diseases^97^ and modulation of mitochondrial metabolism constitutes a key target for emerging anti-fibrotic therapies.^98^ Activation of cGAS–STING signaling in the liver triggers the activation of HSCs and the promotion of a pro-inflammatory milieu^99,100^ and STING is upregulated in patients with metabolic liver disease.^101^ Notably however, other recent studies indicate that the cGAS–STING axis contributes to HSC senescence, which limits hepatic fibrosis.^102,103^ The exact cause underlying these divergent findings is not clear but suggests that the cGAS–STING pathway could play complex context-dependent roles in hepatic inflammation and fibrosis.

In contrast, inflammasome activation has been consistently linked to hepatic inflammatory signaling and fibrosis. Knock-in of a dominant active Nlrp3 in myeloid cells or neutrophils resulted in severe liver inflammation, hepatocyte death and extensive fibrosis.^104,105^ Inversely, the NLRP3 inhibitor MCC950 inhibited hepatic inflammation and fibrosis in a dietary mouse model of MASH.^106^

Combined, the available data suggest that acceleration of DNA glycosylases has beneficial roles for hepatic metabolism, inflammation and fibrosis across model systems. In contrast, the available evidence is less consistent for the role of PARPs and cGAS–STING signaling.

## 5. Facilitated DNA damage repair for pulmonary fibrosis

The lung is continuously exposed to endogenous and exogenous genotoxic stress, including ROS produced during respiration, airborne pollutants, cigarette smoke, infections and mechanical strain. Consequently, epithelial cells, endothelial cells and resident immune cells accumulate DNA damage throughout life. Pulmonary fibrosis emerges when this damage is persistent, poorly repaired, or repeatedly reintroduced, resulting in chronic inflammation, epithelial dysfunction and progressive activation of fibroblasts and myofibroblasts.^107^

Both experimental and clinical studies demonstrate that DNA damage accumulates in fibrotic lung tissue. Elevated levels of oxidative DNA lesions, notably 8-oxoG, but also persistent γH2AX foci and activation of DDR pathways are observed in lung epithelial cells from patients with idiopathic pulmonary fibrosis (IPF) and in bleomycin-induced fibrosis models.^108,109^ These signals trigger apoptosis or senescence in alveolar epithelial cells, impair epithelial regeneration, and generate a pro-fibrotic microenvironment characterized by high levels of TGF-β, IL-1β and other inflammatory mediators.^110^ Importantly, epithelial senescence has emerged as a major determinant of lung fibrosis progression. Senescent alveolar epithelial cells exhibit sustained DDR signalling and establish SASP that promotes fibroblast activation and ECM deposition.^111^ Thus, disease outcome is critically influenced by the ability of lung cells to repair DNA lesions in a sustained fashion.

Oxidative stress is a dominant source of lung DNA damage, making BER a key pathway in pulmonary homeostasis. OGG1-mediated repair of 8-oxoG influences inflammatory gene expression, immune cell recruitment and tissue remodelling in the lung.^112,113^ In acute injury models, pharmacological inhibition of OGG1 using TH5487 reduced pro-inflammatory gene transcription, attenuated fibroblast activation and decreased collagen deposition in bleomycin-treated mice.^114^ These findings provided compelling proof that DNA repair pathways can modulate pulmonary fibrosis. Nonetheless, they are best interpreted in the context of acute injury and early inflammatory signalling.^115^ In such settings, suppressing transcriptional amplification reduces inflammatory cascades that would otherwise accelerate tissue damage. However, follow-up mechanistic studies revealed that OGG1 inhibition stalls repair at the lesion level, prolonging the presence of oxidized bases and repair intermediates.^9,116,117^ While tolerable in the short-term, persistent inhibition under chronic oxidative stress risks the accumulation of lesions, increased replicative stress and epithelial senescence, outcomes associated with progressive fibrosis rather than resolution.

Pulmonary fibrosis in patients is inherently a chronic disease. Repetitive epithelial injury, ongoing oxidative stress, mitochondrial dysfunction and impaired alveolar regeneration create conditions in which DNA damage continuously accumulates. Under these circumstances, suppressing repair activity may transiently reduce inflammatory signalling.^114^ This approach comes at the cost of leaving the underlying genomic damage unresolved.^118^ Evidence from telomere biology further supports this view. Short telomeres and impaired telomere repair are strong genetic risk factors for IPF and telomeric DNA damage in alveolar epithelial cells promotes senescence and fibrotic remodelling.^119^ The Opresko laboratory demonstrated that oxidative base repair at telomeres exacerbates long-term cellular dysfunction, even when acute DNA damage signalling is initially reduced.^120^ These findings argue that an inhibition of DNA damage repair is unlikely to be compatible with long-term lung tissue maintenance.

An emerging alternative strategy is to enhance or rewire DNA repair, enabling lung epithelial cells to clear lesions efficiently and to rapidly terminate DDR signalling before senescence and fibroblast activation arise. Recent studies have demonstrated that OGG1 activity offers the necessary mechanistic plasticity.^121^ Small molecules that act as organocatalytic switches (ORCAs) accelerate processing of AP sites and facilitate BER rather than stalling repair.^122^ While this strategy has not yet been tested in lung fibrosis models, the mechanistic profile observed previously directly addresses the root cause of chronic epithelial damage of inefficient lesion resolution under sustained oxidative load.^92,123^

## 6. Facilitated DNA damage repair for cardiovascular health

Cardiovascular tissues are exposed to continuous mechanical stress, metabolic flux and oxidative injury throughout life. Vascular smooth muscle cells (VSMCs), endothelial cells and cardiomyocytes accumulate DNA damage because of ROS, replication stress and inflammation. Unlike rapidly renewing tissues, cardiovascular cells exhibit limited regenerative capacity.^124^ As a result, failure to adequately repair DNA damage has long-lasting consequences, promoting cellular senescence, clonal dysfunction, inflammation and fibrotic remodelling that together drive cardiovascular disease progression.

Seminal work established that DNA damage induces senescence, which in turn propagates atherosclerosis and vascular aging.^8^ Studies in both human plaques and atherosclerotic mouse models demonstrated accumulation of persistent DNA damage markers, including γH2AX and 53BP1 foci, in VSMCs and endothelial cells. These cells undergo growth arrest and acquire SASP, characterized by secretion of pro-inflammatory cytokines, matrix metalloproteinases (MMPs) and pro-fibrotic factors. Further, VSMC senescence is associated with formation of micronuclei, activation of cGAS–STING and induction of multiple pro-inflammatory cytokines, rendering it a partially mitochondrially controlled process.^125^ The selective removal of senescent cells, or prevention of senescence induction, reduces plaque burden, stabilizes lesions and improves vascular function.^126,127^ These findings place DNA damage upstream of inflammation and fibrosis in cardiovascular disease, highlighting repair capacity as a key determinant of tissue integrity.

Oxidative stress is a dominant source of vascular DNA damage, particularly in regions exposed to disturbed flow or metabolic dysfunction. 8-OxoG accumulates in VSMCs and endothelial cells with age and disease, activating BER and downstream DNA damage response signalling.^128^ As such, persistent BER activity sustains DDR signalling, enforcing senescence and SASP output. Recent work demonstrates that OGG1 activity declines in aging vasculature, coinciding with increased oxidative DNA damage and vessel stiffness.^8^ Importantly, genetic manipulation restoring active OGG1 in VSMCs preserves arterial compliance and prevents age-associated ECM remodelling. These observations support the idea that the efficient resolution of oxidative DNA lesions protects vascular structure and function, while dysfunctional repair accelerates fibrotic remodelling.

Cardiovascular tissues are continuously active and rarely experience discrete, self-limited injury. Since DNA damage accumulates chronically, repair inhibition risks accelerating lesion persistence, senescence and functional decline. Indeed, in VSMCs, DNA repair deficiencies promote clonal expansion of dysfunctional cells, plaque instability and fibrotic vessel wall thickening.^129^ Ample evidence has established that replication stress and unresolved DNA damage in VSMCs drive pathological clonal selection rather than regeneration, impairing vessel integrity and increasing disease risk.^125,130^ Suppressing DNA repair would thus amplify the very processes that drive cardiovascular pathology. In cardiovascular tissues facilitation rather than inhibition of DNA damage repair is therefore the preferred therapeutic strategy. Indeed, restoring repair competence normalizes expression of ECM-regulating proteins such as Lysyl oxidase (LOX) and WNT1-inducible-signaling pathway protein 2 (WISP2) while suppressing TGF-β signalling and collagen accumulation during vascular aging. These molecular changes translate into measurable improvements in arterial compliance and pulse pressure, strengthening the link between DNA repair efficiency and cardiovascular health.

Mitochondrial dysfunction further amplifies cardiovascular disease progression. Damaged mitochondria release oxidized mtDNA, activating cGAS–STING and NLRP3 signalling pathways in both vascular cells and macrophages.^131^ These pathways drive inflammation, endothelial dysfunction and fibrotic remodelling of the vessel wall and myocardium. Because mitochondrial DNA damage arises from chronic metabolic and oxidative stress, mitochondrial repair capacity becomes a second major determinant of cardiovascular health.

## 7. Emerging developments

As discussed above, it emerges that fibrosis is driven less by the magnitude of DNA damage itself than by an imbalance between damage burden and repair capacity over time. When DNA lesions persist, either in the nucleus or the mitochondria, inflammatory signalling is sustained, cellular senescence is promoted and clonal dysfunction intensifies, converging at the initiation of fibrotic tissue remodelling. Emerging therapeutic strategies therefore increasingly focus on facilitating DNA damage repair, rather than on suppressing downstream inflammatory or fibrotic pathways per se. Although several of the approaches discussed above have demonstrated promising effects in mechanistic and preclinical systems, further validation in fibrosis-specific *in vivo* models and clinical settings will be required to establish their therapeutic potential.

Nuclear DNA repair pathways, particularly BER, represent a first layer of therapeutic opportunity. Diseases characterized by episodic or acute oxidative injury, such as acute lung injury or transient toxic exposures, may benefit from temporary inhibition of specific glycosylase activities. In these contexts, glycosylase inhibition can dampen inflammatory transcriptional amplification driven by BER intermediates and PARP-dependent signalling. Importantly, such approaches are likely to be beneficial only if repair capacity is subsequently restored or compensated, avoiding the prolonged persistence of lesions. In contrast, chronic fibrotic diseases, including MASH, IPF and atherosclerosis, are defined by sustained oxidative stress and the continuous accumulation of DNA damage. In these settings, facilitation of DNA repair rather than inhibition becomes essential. Strategies under development include enhancing glycosylase activity with approaches covering expressed enzymes,^49^ activators^132,133^ or ORCAs.^134,135^ The desired benefits for these modalities include increased downstream repair flux, improved chromatin accessibility, maintained NAD^+^ and sirtuin function and the efficient recruitment of repair scaffolds. This is based on recent evidence that sirtuin activity, chromatin plasticity and scaffold availability decline under metabolic stress and with age. As a result, tissues are shifted toward persistent damage signalling, senescence and fibrosis, highlighting the robustness of nuclear repair as both a biomarker and therapeutic target in chronic disease.

In this review, we emphasize oxidative DNA damage and BER because these pathways currently offer the clearest mechanistic and pharmacological opportunities for facilitated repair; however, we also recognize that other lesion classes converge on overlapping pro-inflammatory and pro-fibrotic downstream programs.^136^ As an example, beyond direct modulation of BER enzymes, emerging strategies aim to increase overall somatic DNA repair capacity, for example by targeting transcriptional programs that constrain genome maintenance, such as DREAM complex-dependent repression.^37^

In parallel, increasing attention is focused on the mitochondrial genome as a driver of chronic inflammation and fibrosis.^137^ Accumulating evidence shows that mtDNA damage and leakage into the cytosol convert metabolic stress into innate immune activation, primarily through cGAS–STING and the NLRP3 inflammasome. This positions mitochondrial genome stability as an important mechanistic regulator of fibrotic disease progression. Thus, enhancing mitochondrial DNA repair capacity has emerged as a promising therapeutic strategy. Boosting mitochondrial OGG1 activity could accelerate removal of 8-oxoG lesions, stabilize mtDNA content and support oxidative phosphorylation.^50,52,53^ Similarly, POLG, the sole mitochondrial DNA polymerase, represents a critical bottleneck for both replication and repair. First-in-class small-molecule POLG activators have recently been described and shown to increase mtDNA replication and improve respiratory function in patient-derived cells.^54^ Although fibrosis-specific data are still forthcoming, increased mtDNA copy number and repair capacity should reduce mtDNA fragmentation and release, weaken cGAS–STING activation and attenuate chronic inflammation.

Additional approaches aim to prevent mtDNA release or sensing directly, including pharmacological targeting of cGAS–STING^138^ and NLRP3,^139^ both of which have entered clinical development, as well as improving mitochondrial quality control. Enhancing mitophagy, promoting balanced mitochondrial fusion and fission, stabilizing mitochondrial nucleoids *via* TFAM,^140^ and restoring metabolic cofactors that sustain NAD^+^ (ref. 96 and 141) and SIRT3^142^ activity may maintain mtDNA integrity under oxidative stress.

Evidence from different organs reinforces the generality of these principles. In the skin, facilitated DNA repair has been achieved through topical delivery of lesion-specific repair enzymes, enhancement of endogenous BER, stabilization of telomeres and metabolic support. The strategy has already demonstrated safety and efficacy in reducing inflammation, photoaging and fibrotic remodelling. In the lung, where both nuclear and mitochondrial DNA damage contribute to epithelial senescence and innate immune activation, dual support of nuclear and mitochondrial repair pathways may be required to achieve durable anti-fibrotic effects. In the cardiovascular system, DNA damage-induced senescence is established as a major driver of vascular dysfunction, clonal pathology and fibrosis, positioning dysfunctional DNA repair as a root cause rather than a secondary feature of disease. Across these tissues, enhancing DNA repair capacity acts through conserved, complementary mechanisms that prevent premature senescence by resolving lesions before checkpoint activation, reducing senescence-associated secretory phenotypes and paracrine inflammation, preserving functional cellular fitness and clonal balance and maintaining ECM homeostasis to prevent excessive collagen deposition and tissue stiffening.

Taken together, these findings support a conceptual shift from targeted inhibition to coordinated enhancement of both nuclear and mitochondrial DNA repair. A dual-axis approach that combines improved nuclear BER with stabilization of the mitochondrial genome has the potential to maximize genomic integrity, suppress both nuclear and mitochondrial sources of DAMPs and limit the transition from chronic damage to inflammation, senescence and fibrosis.

As tools to modulate DNA repair with greater precision continue to emerge, future therapeutic development will likely move beyond organ-specific symptom control toward upstream restoration of genome maintenance. Within this therapeutic basis, facilitated DNA damage repair may represent a foundational strategy for preventing and reversing fibrotic disease across tissues.

## 8. Conclusions

Persistent DNA damage has emerged as a central upstream mechanism that links metabolic stress, ageing, chronic inflammation, cellular senescence and ECM remodelling across organ systems. In this paradigm, fibrosis can be seen as the consequence of dysfunctional nuclear and mitochondrial DNA repair processes that fail to effectively resolve DNA lesions in tissues undergoing sustained injury. This perspective shifts therapeutic development away from the ‘simple’ suppression of inflammation and towards the restoration of genome maintenance as a disease-modifying intervention.

Critically, recent advances in human-relevant experimental systems have accelerated the translational viability of this concept. Patient-derived 3D organotypic and microphysiological cultures now allow for the scalable and direct interrogation of DNA damage repair dynamics in disease-relevant human contexts.^143^ These systems capture key features of tissue fibrosis, such as metabolic stress, mitochondrial dysfunction, pro-inflammatory signaling, cellular senescence and ECM remodeling, and enable quantitative assessments of the flux through DNA damage repair pathways in response to chemical modulation.^144–146^ As such, they provide an appealing translational approach that can efficiently test new chemical series and shortlist promising lead candidates, thereby bridging between chemical biology and therapeutic development.

From an industry perspective, DNA damage repair pathways represent a largely untapped space for the development of novel modalities against chronic inflammatory and fibrotic diseases with high first-in-class potential. Particularly, DNA glycosylases offer defined catalytic activities and druggable interfaces that can be selectively tuned rather than globally inhibited. Chemical biology strategies that facilitate repair, such as ORCAs or mitochondrially targeted facilitators of DNA damage repair, enable the suppression of DAMPs, thereby acting upstream of immune activation, cytokine release and fibroblast transdifferentiation. Based on the available evidence and developmental trajectory, we foresee that facilitation of DNA damage repair is poised to become an attractive, clinically applicable strategy for chronic inflammatory and fibrotic diseases across organ systems in the near future.

## Conflicts of interest

VML is CEO and shareholder of HepaPredict AB, as well as co-founder and shareholder of Shanghai Hepo Biotechnology Ltd. The other authors declare no competing interests.

## Data Availability

This work is a Review article. As such, no original data was generated.
